# Hemodynamic and Systemic Effects of Albumin in Patients with Advanced Liver Disease

**DOI:** 10.1007/s11901-020-00521-1

**Published:** 2020-07-01

**Authors:** Manuel Tufoni, Maurizio Baldassarre, Giacomo Zaccherini, Agnese Antognoli, Paolo Caraceni

**Affiliations:** 1 Department of Medical and Surgical Sciences, S. Orsola-Malpighi University Hospital, Alma Mater Studiorum University of Bologna, Via Albertoni 15, 40138 Bologna, Italy; 2 Center for Applied Medical Research (CRBA), Alma Mater Studiorum University of Bologna, Bologna, Italy

**Keywords:** Liver cirrhosis, Albumin, Circulatory dysfunction, Systemic inflammation, Ascites

## Abstract

**Purpose of Review:**

Albumin administration is recommended to prevent or treat specific complications of decompensated cirrhosis based on its capacity to expand plasma volume. However, the molecule also has many other biological properties that are unrelated to the oncotic activity. The purpose of this review is to examine the hemodynamic and systemic effects of albumin administration in patients with decompensated cirrhosis.

**Recent Findings:**

Besides plasma expansion, albumin appears to act against inflammation, facilitate immunocompetence, and improve cardiac and endothelial function, thus antagonizing critical steps in the pathophysiological cascade underlying decompensated cirrhosis.

**Summary:**

Increasing knowledge of the pathophysiological mechanisms of the disease, as well the pleiotropic properties of the molecule, provides the rationale for considering albumin as a multi-target disease-modifying agent in decompensated cirrhosis. Both oncotic and non-oncotic properties likely concur with the clinical benefits of long-term albumin administration recently demonstrated in these patients.

## Introduction

Human albumin is the most abundant protein circulating in the blood and has many biological functions. The administration of exogenous albumin started in World War II when it was used for fluid resuscitation in traumatic shock. Treatment with albumin has been extended to many other pathological conditions, despite the fact that its efficacy has either been disproved by evidence-based medicine or is still under debate.

Hepatology is an area where the prescription of albumin is recommended by international guidelines. In fact, randomized clinical trials and metanalyses have demonstrated its efficacy in preventing or treating several severe complications of cirrhosis, i.e., prevention of post-paracentesis circulatory dysfunction (PPCD) and renal failure after spontaneous bacterial peritonitis (SBP), and the diagnosis and treatment of hepatorenal syndrome (HRS) [1••, 2•, 3•] (Table 1).

**Table 1:** Summary of the current indications for the use of human albumin in patients with cirrhosis. Adapted from Caraceni et al. [3•]

Clinical condition		Doses and administration schedules	Indication
Prevention of PPCD	Paracentesis ≥ 5 l	6–8 g per liter of removed ascites	Mandatory in all patients
	Paracentesis < 5 l		Preferred due to concerns regarding use of synthetic colloids or crystalloids
Prevention of renal failure after SBP	High-risk patients	1.5 g/kg b.w. at diagnosis + 1 g/kg b.w. on the 3rd day	Mandatory in all patients
	Low-risk patients* = serum bilirubin <4 mg/dL and serum creatinine <1 mg/dL		Consider in individual patients
Diagnosis of HRS		1 g/kg/ b.w. per day for 2 consecutive days	To be used regularly
Treatment of type I HRS (in association with vasoconstrictors)		1 g/kg b.w. at diagnosis plus 20–40 g/day until vasoconstrictors are stopped	Mandatory in all patients
Treatment of severe hyponatremia		To be defined	Consider if no response to standard measures
Prevention of renal failure after non-SBP bacterial infections		------	Not indicated at present
Treatment of septic shock		To be defined	Consider in all patients
Treatment of hepatic encephalopathy		------	Not indicated at present

*PPCD*, post-paracentesis circulatory dysfunction; *SBP*, spontaneous bacterial peritonitis; *HRS*, hepatorenal syndrome

Two international surveys and one national survey have shown that hepatologists prescribe albumin, although not on a regular basis, also for other acute conditions not supported by solid scientific evidence, such as non-SBP bacterial infections, hepatic encephalopathy, hyponatremia, and muscle cramps [4–6]. Finally, clinical trials have also assessed the efficacy of long-term albumin administration in patients with cirrhosis and ascites, opening up new perspectives for its use in decompensated cirrhosis [7••, 8•, 9•].

Until a few years ago, the clinical effects of albumin were attributed almost exclusively to its capacity to expand plasma blood volume, thus counteracting the effective hypovolemia and the related hemodynamic alterations that characterize advanced cirrhosis. However, in the last decade, experimental and clinical evidence has highlighted that the benefits of albumin are also mediated by systemic effects resulting from the non-oncotic properties of the molecule, which are closely related to the integrity of its structure.

## Structure and Function of the Albumin Molecule

Human albumin is a water-soluble monomeric protein consisting of 585 amino acid residues with a molecular weight (MW) of 66,438 Da [10]. The primary sequence of the protein contains one residue of tryptophan (Trp214) and several charged amino acid residues (lysine, arginine, glutamic and aspartic acid), which confer a negative net charge to the protein at physiological pH, thus making albumin a highly water-soluble molecule [11, 12]. The albumin structure is composed of three homologous domains (I, II, III), each of which contains 10 α-helices and is divided into two subdomains (A and B). Albumin contains 35 cysteine residues, 34 of which are involved in the formation of 17 disulfide bonds, which contribute to protein stability and flexibility. In solution, albumin takes on a globular heart-shaped conformation, although physico-chemical studies have revealed that it changes its shape easily [13, 14].

Albumin is exclusively synthetized by hepatocytes and released into the circulation at a rate of about 13–14 g per day under physiological conditions. However, only about a third of the total albumin pool remains in the vascular compartment, while the rest is found in the interstitial fluids or other organs, such as the skin, muscles, and gut [15]. As a result, the serum albumin concentration in a healthy adult ranges between 3.5 and 5.0 g/dl, thus representing more than 50% of the total plasma proteins and by far the most abundant circulating protein in the body [15]. Its persistence in the blood is also favored by constant uptake and recycling by hepatocytes leading to a relatively long total half-life of about 16–20 days [15], which can, however, be shortened by enhanced catabolism and clearance in the case of structural alterations to the molecule [16], as occurs during aging or acute and chronic disease states.

Both the particular molecular structure and the high concentration in the blood constitute the basis for the involvement of albumin in the regulation of a wide range of physiological processes.

Albumin is the main modulator of fluid distribution among body compartments, accounting for about 70–80% of the plasma oncotic pressure. The oncotic property of albumin derives from the osmotic effect directly related to its molecular mass (about 2/3) and from the Gibbs-Donnan effect (about 1/3). The latter consists in the capacity of albumin, which is negatively charged, to attract positively charged molecules, such as sodium, thus inducing the shift of water from the extravascular into the intravascular compartment [15]. As a result, albumin has been used for decades as a plasma expander in pathological conditions characterized by the presence of hypovolemia.

Albumin also has several other biological functions, such as binding, transport and detoxification of many endogenous and exogenous compounds, antioxidant activity, modulation of inflammatory and immune responses, stabilization of endothelia, and regulation of coagulation and platelet function [17]. Such properties have been defined as non-oncotic since they are directly related to the conformation and structure of the molecule rather than its oncotic power.

Albumin binds a long series of molecules, including bilirubin, bile acids, fatty acids, hormones, drugs, and drug metabolites, thus increasing their plasma solubility and their transport to the site of action or elimination [18]. The albumin molecule contains seven binding sites for long-chain non-esterified fatty acids [19], and three high-affinity binding sites, namely Sudlow’s sites I and II [20], which are in subdomains IIA and IIIA, and a third binding site recently characterized at subdomain IB [21]. However, many binding sites have still not been identified and their function remains undetermined.

The antioxidant activity of albumin is mainly attributable to the presence of a free cysteine residue at position 34 (Cys-34), which represents the largest plasma source of free thiols, and therefore the most important circulating scavenger of reactive oxygen and nitrogen species [22]. According to the redox state of the Cys-34, three major isoforms of albumin can be found under physiological conditions: (i) in 70–80% of albumin, Cys-34 is in the reduced form and is able to fully exert its activity (mercaptalbumin [HMA]); (ii) in 20–30% of albumin, Cys-34 is inactivated as it is reversibly bound to various small thiol compounds (non-mercaptalbumin 1 [HNA-1]); and (iii) in less than 5% of albumin, Cys-34 is irreversibly oxidized to sulfinic or sulfonic acid (non-mercaptalbumin 2 [HNA-2).

While the function of the Cys-34 residue is permanently lost in HNA-2, it can be restored in HNA-1, as this reversibly oxidized isoform is in a dynamic equilibrium with the reduced isoform HMA [22]. Also, two additional albumin sites, namely the multi metal binding site or site A, located at the interface of domain 1 and domain 2, and the N-terminal portion of the molecule, provide additional indirect antioxidant activity by binding transition metal ions, such as copper, nickel, and cobalt, thus inhibiting their capacity to catalyze many chemical reactions, in turn generating free radicals [23].

The role of albumin as a modulator of inflammatory and immunological responses has attracted increasing research interest. Some studies indicate that albumin interferes with the immune and inflammatory activation by modulating the redox state and the bioavailability of many molecules involved in these processes. Examples of inflammatory activation are the binding of bacterial products (e.g., lipopolysaccharide) [24, 25] or prostaglandin E2 (PGE_2_) [26].

However, more recent evidence shows that, after being internalized in the immune cells via early endosomes, albumin also reduces the production of cytokines by blocking TLR3 and TLR4 signaling [27]. In addition, albumin can be actively involved in the host defense response: one example is its capacity to bind the *Clostridium difficile* toxin at domain II in an experimental model of zebrafish embryos, so that albumin prevents the internalization of the toxin within the host cells [28].

Finally, albumin contributes to the vascular integrity by binding the endothelial extracellular matrix, thus modulating the transcapillary fluid exchange [29]. It also antagonizes endothelial dysfunction by blunting the oxidative and nitrosative stress as well as cell adhesion and the inflammatory response in endothelial cells [30, 31].

## Effects of Albumin Administration in Advanced Cirrhosis

Exogenous albumin has been administered for decades in patients with decompensated cirrhosis in order to expand the plasma blood volume. It is now clear that albumin administration has many other effects unrelated to its oncotic power. For the sake of simplicity, the hemodynamic and systemic effects will be reviewed separately; however, it should be clearly stated that this is an artificial distinction since the net result is the sum of both effects, which often interact with each other.

### Circulatory Dysfunction

The circulatory dysfunction secondary to portal hypertension consists of a reduction in systemic vascular resistance due to arterial vasodilation, above all in the splanchnic circulation, leading to effective hypovolemia [32]. This splanchnic vasodilation is due to an imbalance in vasoactive substances with the predominance of those with vasodilating activity (i.e., nitric oxide, endogenous cannabinoids, and carbon monoxide) [33–35]. Effective hypovolemia, which is sensed by the baroceptors, activates vasoconstrictor and sodium-retaining pathways (renin-angiotensin-aldosterone system, sympathetic nervous system, vasopressin), which lead to a compensatory increase in cardiac output.

With the progression of the disease, the worsening of effective hypovolemia cannot be counterbalanced by a further increase in cardiac output despite the intense activation of the compensatory neuro-hormonal systems, thus inducing a reduced organ perfusion and promoting the onset of severe cirrhosis complications [32]. In the more advanced stages of the disease, an impairment in cardiac function also contributes to the extent of effective hypovolemia, as witnessed by the decrease in the cardiac index from compensated cirrhosis to decompensated cirrhosis and hepatorenal syndrome [36–38].

### Hemodynamic Effects of Albumin Administration

Based on this pathophysiological scenario, effective hypovolemia represents a logical target of treatment in decompensated cirrhosis. The administration of albumin has proven to be more effective than other fluids in achieving this goal, so that it has become a cornerstone for the prevention of PPCD or renal failure after SBP, and treatment of HRS [1••, 2•, 3•].

PPCD is diagnosed by an increase in plasma renin activity (PRA), a consolidated marker of effective hypovolemia, of over 50% after 4–6 days from large-volume paracentesis. PPCD is associated with arterial hypotension, renal dysfunction and hyponatremia, more frequent re-hospitalizations, and reduced survival [39]. When more than 5 l of ascites is removed, randomized trials and meta-analyses have shown that albumin administration (8 g per each liter of ascites removed) is superior to other plasma expanders (crystalloids and synthetic colloids) or vasoconstrictors in antagonizing the increase in PRA, and reducing the incidence of complications related to circulatory dysfunction [40–43, 44•].

In the context of SBP, the administration of albumin (1.5 g/kg b.w. at diagnosis and 1 g/kg b.w. on day 3) significantly reduces the development of renal failure and the in-hospital and 3-month mortality [45•]. Fernandez et al. demonstrated that albumin, but not hydroxyethyl starch, succeeds in lowering PRA and increasing mean arterial pressure [46•]. Even more interestingly, this study, published in 2005, showed that the albumin-induced improvement of systemic hemodynamics in patients with SBP was not attributed to plasma expansion alone. In fact, the increase in arterial pressure was related not to a higher cardiac index but to a striking increase in peripheral vascular resistance. This was probably due to the attenuation of endothelial activation, as witnessed by the significantly reduced plasma levels of Factor VIII and von Willebrand–related antigen observed in patients receiving albumin, but not in those treated with hydroxyethyl starch [46]. However, the hemodynamic measurements were performed days apart and the resolution of sepsis may have theoretically facilitated the increase in the vascular tone.

Similar data on systemic hemodynamics have been observed in patients presenting bacterial infection other than SBP. Guevara et al. showed that albumin (1.5 g/kg b.w. at diagnosis and 1 g/kg b.w. on day 3) is able to reduce PRA, plasma aldosterone, and norepinephrine concentrations, but increases the circulating levels of atrial natriuretic peptide [47]. The improvement in circulatory dysfunction and effective hypovolemia in non-SBP bacterial infection were confirmed very recently by the INFECIR-2 study, which showed that albumin, at the same dosage as reported in the previous studies, increased mean arterial pressure and lowered PRA [48•]. Unlike patients with SBP, however, there appears to be no clear clinical advantage in terms of both renal failure and survival in patients with non-SBP bacterial infections. These complications are thus not included among the accepted indications for albumin administration in decompensated cirrhosis [1••, 2•, 3•]. Furthermore, the development of pulmonary edema has only been reported in a few patients [48•, 49]. Thus, caution should be taken in administering high doses of albumin in those patients who are at risk of increased capillary permeability and volume overload (i.e., heart failure, severe pneumonia).

Albumin is also effective in expanding plasma volume in patients with HRS. In the study by Ortega [50], comparing terlipressin plus albumin (1 g/kg b.w. on the first day followed by 20–40 g per day) versus terlipressin alone, the use of albumin was associated with increased arterial pressure, central venous pressure (CVP), atrial natriuretic peptide levels, and suppression of the renin-aldosterone and sympathetic nervous systems, together with a notable decrease in serum creatinine. In contrast, no significant differences were observed in patients who received only terlipressin, except for a reduction in plasma norepinephrine levels [50•].

Almost all studies have described the hemodynamic effects of albumin administered according to fixed schedules and dosages, which were determined quite arbitrarily by the researchers. Very few data are available on guiding albumin administration according to the hemodynamic response. In a trial comparing noradrenalin and terlipressin in patients with HRS, albumin infusion was titrated to achieve a central venous pressure (CVP) between 10 and 15 cm H_2_0, as estimated using a central venous line [51•]. One of the most interesting findings was the heterogeneity among patients in the amount of albumin needed to maintain an adequate CVP, with variations close to 100%, regardless of the type of vasoconstrictor used [51•]. In another small study performed in an intensive care unit, 14 patients with HRS type I received up to 400 ml of 20% albumin solution every 12 h for 48 h under hemodynamic monitoring by transpulmonary thermodilution [52]. The median albumin infused per day was particularly high, i.e., 1.6 g/kg/b.w. (range 1.5–2.0 g/kg/b.w.), which induced HRS reversal without vasoconstrictors in eight patients. However, one or more doses of albumin had to be withheld because of dyspnea or signs of fluid overload in five patients. The hemodynamic effect of albumin infusion was compatible with a preload-driven increase in cardiac index and stroke volume index associated with a decrease in systemic vascular resistance index, PRA, and aldosterone, suggesting that reduced vasoconstriction and, consequently, afterload may have contributed to the increase in cardiac index [52].

The cardiovascular changes induced by albumin treatment in patients with decompensated cirrhosis have also been recently studied during long-term administration.

The Pilot-PRECIOSA study [53••] analyzed the effects of albumin infusion at two different doses for 12 weeks in patients with ascitic cirrhosis and evidence of circulatory dysfunction: 10 patients received 1 g/kg b.w. of albumin every 2 weeks (low-albumin dose group), while 8 patients received 1.5 g/kg b.w. every week (high-albumin dose group). Although neither doses led to a steady suppression, only the high-albumin dose was able to significantly reduce the variability in PRA levels, suggesting a stabilization of circulatory function. Moreover, treatment with the high-albumin dose, but not with the low-albumin dose, was associated with a significant increase in cardiac index, systolic volume, and left ventricular stroke work index, indicating an improvement in the overall cardiac function. In contrast, there was no change in the cardiac preload, as witnessed by the absence of changes in atrial pressure, pulmonary capillary wedged pressure, and plasma concentrations of atrial and brain natriuretic peptides. Finally, no significant changes in portal pressure were observed even with the high-albumin dose [53••].

The results of the Pilot-PRECIOSA study indicated that the amount of albumin infused matters. Additional indirect evidence supporting this hypothesis derives from the results of the placebo-controlled “Midodrine-albumin in cirrhotic patients awaiting liver transplantation” (MACHT) trial [8•]. In this study, which assessed the effects up to one year of a much lower dose of albumin (40 g every 15 days) plus the α1-receptor agonist midodrine, only a mild and transient decrease in the activities of the renin-aldosterone and sympathetic nervous systems was observed without any significant improvement in patient clinical outcome [8•].

Taken together, all this evidence indicates that in patients with decompensated cirrhosis: 1) albumin administration induces significant hemodynamic changes both after acute, short-term and chronic, long-term administration; 2) the common feature is an improvement in effective hypovolemia; 3) besides plasma expansion, indirect evidence supports the hypothesis that the non-oncotic properties of the molecule contribute to this effect by acting on the cardiac and endothelial function; 4) some hemodynamic effects of albumin appear to be transient and are only measurable for a short time after the infusion, such as the increase in central blood volume, while others seem to be more durable and persistent during long-term albumin therapy, such as the improvement in cardiac function and the stabilization of circulatory function; and 5) although a direct correlation with albumin dosage cannot be demonstrated, a pre-requisite for the achievement of significant effects appears to be the administration of a sufficient amount of albumin.

### Systemic Inflammation

Although hemodynamic alterations account for the development of many clinical manifestations, they cannot fully explain the multiorgan dysfunction and failure developing during the course of decompensated cirrhosis [54••]. It is now well accepted that the systemic spread of pathogen-associated molecular patterns (PAMPs), due to abnormal translocation of bacterial products from the gut as a result of a series of alterations in the intestinal barrier initiated by portal hypertension [55], and due to damage-associated molecular patterns (DAMPs), released by the diseased liver [56], activate immune cells through binding with innate recognition receptors. Activated immune cells produce pro-inflammatory cytokines and molecules, along with reactive oxygen and nitrogen species, thus inducing a pro-inflammatory and pro-oxidant internal milieu [54], and contributing to the immune dysfunction observed in patients with advanced cirrhosis [57]. This sequence of events leads to the development of organ failure and dysfunction by: 1) inducing the synthesis of vasoactive substances (i.e., nitric oxide, carbon monoxide, prostacyclin and endocannabinoids), which causes arterial vasodilation and therefore effective hypovolemia [54]; 2) provoking cell and tissue damage due to an excessive inflammatory response (known as immunopathology) [58•]; and 3) affecting cell metabolism characterized by an energetic crisis due to mitochondrial dysfunction [59••]. The degree of systemic inflammation intensifies in parallel with the severity of cirrhosis, reaching a maximum in patients with acute-on-chronic liver failure including patients with the highest degrees of severity [54, 60••].

### Systemic Effects of Albumin Administration

The systemic effects of albumin are mainly related to its many non-oncotic properties, which theoretically could antagonize several pathogenic processes of systemic inflammation and immune dysfunction.

The first indirect evidence of the role of non-oncotic properties derives from the observation that, in patients with SBP, the increase in arterial pressure is mediated, at least in part, by the improvement in endothelial dysfunction following albumin administration [46•], as previously discussed. A few years later, an ex-vivo experimental study on hearts isolated from cirrhotic rats demonstrated that albumin, but not hydroxyethyl starch, improves left cardiac contractility, irrespectively of volume expansion, by blunting oxidative stress and the activation of the nuclear factor kappa-light-chain-enhancer of activated B cells (NF-kB) which, in turn, results in a lower production of tissue tumor necrosis factor-alpha (TNF-α) [61].

A mechanism of immunosuppression in patients with advanced cirrhosis is the increased level of circulating PGE_2_ [62•]. PGE_2_ is a bioactive lipid endowed with a paradoxical effect on the immune and inflammatory response: although PGE_2_ drives the acute inflammatory response, it also promotes immunosuppression during chronic inflammation [63], which is a distinctive feature of patients with advanced cirrhosis [57, 60]. These patients are characterized by high free PGE_2_ plasma levels which are due to an increased synthesis of the lipid mediator as well as from its increased bioavailability due to the reduced amount of albumin. Supporting this hypothesis, monocyte-derived macrophages (MDM) incubated with plasma samples from patients with acute decompensation of cirrhosis elicited a blunted TNF-α production following lipopolysaccharide (LPS) stimulation. Interestingly, this effect was not observed when the same plasma samples were supplemented in vitro with albumin up to a concentration of 40 mg/dl [62•]. The same effect was also observed when albumin was administered in vivo*.* In fact, LPS-stimulated MDM, incubated with plasma samples from patients receiving 20% albumin solution, showed a significantly increased TNF-α production compared to the same cells incubated with plasma samples collected from the same patients before albumin treatment [64].

A clear anti-inflammatory effect of albumin administration was recently shown in the previously mentioned Pilot-PRECIOSA and INFECIR-2 studies through the sequential measurement of markers of systemic inflammation and cytokines [53••]. In the Pilot-PRECIOSA study [53••], conducted on 18 non-infected patients with cirrhosis and severe circulatory dysfunction, the high albumin dose (1.5 g/kg b.w. per week) showed a significant effect on the plasma levels of IL-6, a paradigmatic proinflammatory cytokine, compared to patients treated with the low albumin dose (1 g/kg b.w every two weeks). This finding prompted the authors to perform an additional wider analysis, in which a panel of 24 cytokines was measured in the bio-banked sera from 15 patients with cirrhosis receiving albumin and from 25 healthy controls. As expected, the cytokine circulating levels were higher in patients with cirrhosis than in healthy controls. Interestingly, treatment with albumin was associated with a marked reduction in most cytokine concentrations, but only in the group receiving the high albumin dose [53••].

Consistent results were provided by the measurement of plasma cytokine levels from patients with bacterial infections unrelated to SBP included in the INFECIR-2 study [48•]. The core analysis showed a significant reduction in the white blood cell (WBC) count, C-reactive protein (CRP) and IL-6 in the antibiotics plus albumin arm, while patients treated with antibiotics alone only presented a reduction in CRP [48•]. In a subsequent analysis performed on 78 bio-banked sera and considering the same panel of 24 cytokines measured in the Pilot-PRECIOSA study, only patients from the experimental arm showed a significant decrease in most cytokines, indicating that albumin could play a role in attenuating systemic inflammation related to bacterial infections [53••].

Interestingly, in the same two series of patients, a broad variety of soluble factors (i.e., chemokines, growth factors, markers of macrophage activation and endothelial dysfunction) were measured but albumin treatment was not associated with significant changes in most of them, thus suggesting an effect mainly directed at the regulation of specific cytokines.

These results apparently conflict with the results from the MACHT Trial [8•], in which albumin administration plus midodrine did not affect the plasma levels of IL-1b, IL-6 and TNF-a compared to placebo in patients with decompensated cirrhosis. However, as noted above in terms of the hemodynamic effects, the amount administered in the high albumin dose group of the pilot-PRECIOSA study was three to four times higher than the dose given in the MACHT trial, thus supporting the hypothesis that a sufficient dosage of albumin should be infused in order for there to be benefits.

Taken together, these data highlight the clear anti-inflammatory effect of albumin. Nonetheless, experimental evidence suggests that albumin could act as a “double-face” protein in modulating the inflammatory response.

In fact, the non-oncotic functions of albumin are strictly dependent on its molecular composition and structure. However, in many chronic and acute disease conditions, including advanced cirrhosis, the albumin molecule undergoes several post translational modifications, probably as a result of the proinflammatory and prooxidant microenvironment [16] (Table 2). In this setting the amount of albumin in its native form, characterized by a fully preserved structure, is dramatically reduced, with its relative amount being almost halved with respect to healthy individuals [65•, 66•].

**Table 2:** Structural alterations of the albumin molecule detected in plasma samples by high-performance liquid chromatography coupled to electrospray ionization mass spectrometry. Isoforms belong to the mercaptalbumin (HMA—reduced form), non-mercaptalbumin-1 (HNA-1—reversibly oxidized form) or non-mercaptalbumin-2 (HNA-2—irreversibly oxidized form) fraction according to the redox state of the cysteine 34 residue

Isoforms	Structural alteration	Cys-34 redox state
HSA-DA	N-terminal truncated (-Asp-Ala)	Reduced
		HMA
HSA-L	C-terminal truncated (-Leu)	Reduced
		HMA
HSA+CYS-DA	Cysteinylated and N-terminal truncated (-Asp-Ala)	Reversibly oxidized
		HNA1
HSA (DHA)	Conversion of a cysteine into dehydroalanine (DHA)	Reduced
		HMA
Native HSA	Native form of HSA	Reduced
		HMA
HSA+SO_2_H	Sulfinylated	Irreversibly oxidized
		HNA2
HSA+SO_3_H	Sulfonylated	Irreversibly oxidized
		HNA2
HSA(DHA)+CYS	Conversion of a cysteine into DHA and cysteinylated	Reversibly oxidized
		HNA1
HSA+CYS	Cysteinylated	Reversibly oxidized
		HNA1
HSA+GLY	Glycosylated	Reduced
		HMA
HSA+CYS+GLY	Cysteinylated and glycosylated	Reversibly oxidized
		HNA1

In contrast, damaged albumin isoforms accumulate in the vascular compartment, as witnessed by the significant amounts of HNA1 and HNA2 commonly found in patients with cirrhosis [60••]. In addition, reflecting the systemic redox state, oxidized albumin isoforms actively contribute to the systemic inflammatory response. In fact, HNA1, which includes all the reversible oxidized albumin isoforms at the Cys34 level, triggers cytokine production in human leucocytes by activating the p38 mitogen-activated protein (MAP) kinase pathway [67•]. HNA1 also induces the production of eicosanoids, such as PGE_2_, which in turn promote immunosuppression in patients with advanced cirrhosis [62•, 67•]. In cases of severe alcoholic hepatitis, a condition characterized by significant oxidative and non-oxidative damage to the albumin molecule [68], it has been shown that HNA2, which includes all albumin isoforms with irreversible oxidation of the Cys34 residue, promotes neutrophils [69] and platelet activation through a CD36 mediated mechanism [70], leading to the release of proinflammatory and prooxidant mediators.

Overall, these in vitro data suggest that the immunomodulatory activity of albumin is profoundly influenced by its redox state. Albumin could thus act as a proinflammatory and prooxidant agent when oxidized, while it can counteract the systemic inflammatory response when predominantly reduced.

All this evidence indicates that in patients with decompensated cirrhosis: 1) systemic effects related to the non-oncotic properties of the molecule probably contribute to the benefits seen after acute and chronic albumin administration; 2) modulation at different steps of the inflammatory and immunological responses appears to be the main systemic effect of albumin in patients with both “sterile” systemic inflammation and infection-related inflammation; 3) the degree of the anti-inflammatory activity of albumin may be related to the preserved molecular structure, thus highlighting the possibility of improving the quality of commercial albumin preparations as a therapeutic option; and 4) as for the hemodynamic effects, the amount of infused albumin appears to play a role in determining the impact of the systemic effects.

## Conclusions: Clinical Impact, Practical Considerations, and Future Perspectives

**Fig. 1:**
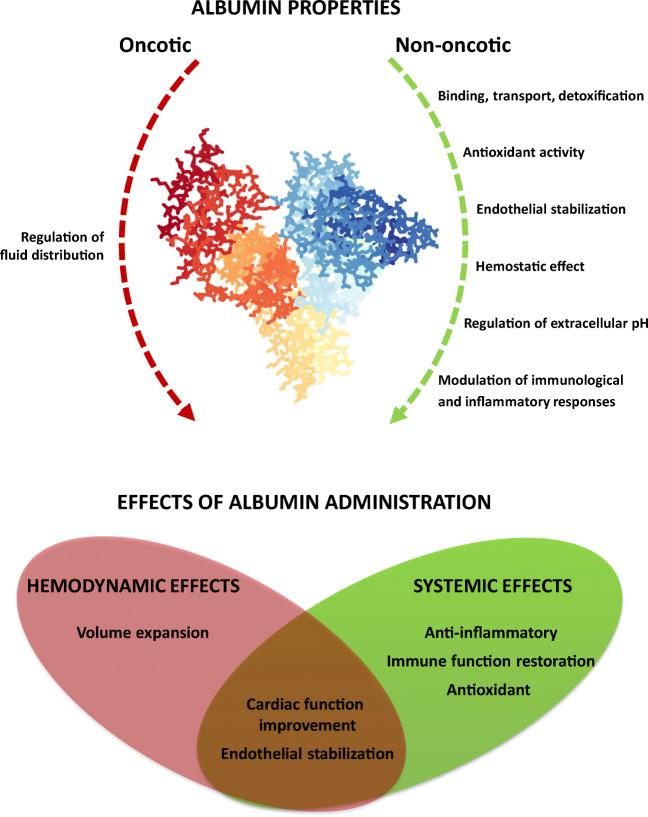
Oncotic and non-oncotic properties of the albumin molecule and effects of exogenous albumin administration in patients with decompensated cirrhosis. Albumin image from the RCSB PDB (rcsb.org) of PDB ID 1AO6 (Sugio et al. [14])

Albumin has been used for the last three decades to prevent or treat specific complications in patients with cirrhosis and ascites based on its capacity to expand the plasma volume. However, in the last few years, increasing knowledge of the pathophysiological mechanisms underlying decompensated cirrhosis, as well as the pleiotropic properties of the molecule, provides the rationale for considering albumin as a multi-target disease-modifying agent for these patients (Fig. 1). In this context, the ANSWER trial [7••], is a non-profit, open-label, randomized, multicenter clinical trial, performed on patients with cirrhosis and at least grade 2 non-complicated ascites, despite an ongoing diuretic treatment of at least 200 mg per day of an anti-aldosteronic drug plus at least 25 mg per day of furosemide. The results of this trial have shown that long-term albumin administration, in addition to easing the management of ascites, prolongs survival, reduces the incidence of life-threatening complications, and the frequency and length of hospitalizations, and also improves the quality of life and appears to be cost-effective [7••]. Based on these results, long-term albumin administration may become the first disease-modifying agent in patients with advanced cirrhosis.

Besides the need for confirmatory scientific data from ongoing clinical trials (ATTIRE, PRECIOSA, APACHE) that enroll patients at different stages of decompensated cirrhosis, several practical aspects should also be addressed in order to implement long-term albumin administration in daily clinical practice. In fact, potential barriers to the widespread adoption of chronic albumin administration include inadequate healthcare facilities, patients’ transportation or work activities, or patients’ willingness to undergo the treatment and caregivers’ willingness to help them.

In our experience, the administration of four 50 cc 20% albumin vials (40 g) requires about one hour from patient arrival and administrative acceptance in our Liver Outpatient Clinic to discharge, with one nurse able to manage up to 3–4 patients sitting on infusion chairs almost at the same time. For the few patients with logistical problems (i.e., related to the distance from hospital or their caregiver’s willingness to accompany them) or events producing restrictions to hospital access (e.g., viruses such as COVID-19), we adapt schedules and doses of albumin by prolonging the interval between infusions and increasing the dose administered at each infusion to 60 g every 10 days, or less frequently to 80 g every 14 days. We do not usually use the higher dosage of 80 g with patients presenting concomitant evidence of heart failure due to the risk of volume overload or patients with high-risk esophageal varices, even though the ANSWER trial did not show differences in variceal bleeding at the standard dose of 40 g weekly [7••].

Changing the approach to albumin treatment, from a mere plasma expander targeting acute conditions to a disease-modifying therapy performed through long-term infusions, would entail an organizational shift from a hospital-centered toward a territorial-based model. In the experience of many centers throughout Italy, patients with cirrhosis and ascites often receive albumin treatment in the health-care centers closest to where they live or even at home, thus limiting the need to access hospital facilities. This type of organization also facilitates collaborations between hospitals and local services, hopefully optimizing the care of patients and the use of health-care resources.

Finally, in our experience, nearly all patients appear to be strongly committed to adhere to this chronic treatment, even though it is demanding, since they perceive a clear health benefit. Notably, in the ANSWER trial, adherence to albumin therapy was higher than 90% [7••].

The implementation of this new strategy in daily clinical practice would also be enhanced by considering using the serum albumin concentration to guide treatment or identifying guidelines for stopping treatment.

Interestingly, a post hoc analysis of the ANSWER trial [71] showed that the serum albumin levels reached after 1 month of treatment were strongly associated with the probability of 18-month survival, which was greater than 90% in those patients reaching a level of 4 g/dl. It could be argued that this value is quite a bit above the lower limit of the normal range of serum albumin concentration (3.5–5.0 g/dl). However, healthy individuals with a comparable age to those patients included in the ANSWER trial present a median serum albumin concentration of 4.2–4.3 g/dl, with about 90% of them above the value of 4.0 g/dl [72] Thus, as with many other biochemical parameters, the “true” healthy serum albumin concentration appears higher than the level corresponding to the lower limit of the range of normality indicated by lab reports.

In the ANSWER trial, the median serum albumin concentration at 1 month of treatment was 3.7–3.8 g/dl, which was maintained during the rest of the study, and about one-third of patients reached the target of 4.0 g/dl [7••]. Baseline serum albumin concentration and baseline MELD independently predicted the achievement of this threshold level [71]. Based on these data, our current approach, particularly in patients with very advance disease and severe hypoalbuminemia, is to extend the period of the loading dose used in the ANSWER study (40 g twice a week for the initial 2 weeks) until the serum albumin concentration reaches levels at least close to 4 g/dl.

It could be also argued that albumin treatment is futile if the serum albumin concentration does not reach at least the threshold level of 3.5 g/day. However, this is not the case because the 40 patients of the ANSWER trial treated with albumin and remaining hypoalbuminemic after one moth of treatment were more likely to survive than those receiving only diuretics (personal communications).

Conversely, there are still no criteria indicating when therapy should be stopped. From an initial retrospective assessment of our cohort of patients, albumin administration is usually maintained until transplantation in decompensated wait-listed patients, and until or close to death in patients with end-stage liver disease not amenable to transplantation since they had significant advantages from treatment based on the results of the ANSWER trial [7]. However, in patients who have steadily mobilized ascites and whose clinical conditions remain stable for weeks, in our clinic we first reduce the frequency of administration and then attempt to stop treatment. Termination is then followed by clinical and laboratory monitoring for early identification of any patients that need to resume albumin administration. With this approach, we have managed to stop albumin infusion in at least 20–30% of patients, particularly in those receiving etiological treatments (personal communication). Future studies are eagerly awaited to define this important issue.

Finally, in addition to clinical studies, translational research is also needed to better clarify the clinical impact of the structural and functional alterations of albumin that occur in patients with advanced cirrhosis. This, in turn, may provide the rationale for identifying novel biomarkers of prognosis and treatment response as well as for the development of improved commercial albumin formulations.
